# *Plasmodium* serine hydroxymethyltransferase: indispensability and display of distinct localization

**DOI:** 10.1186/1475-2875-11-387

**Published:** 2012-11-22

**Authors:** Wichai Pornthanakasem, Darin Kongkasuriyachai, Chairat Uthaipibull, Yongyuth Yuthavong, Ubolsree Leartsakulpanich

**Affiliations:** 1 National Center for Genetic Engineering and Biotechnology, 113 Phahonyothin Road, Khlong Nueng, Khlong Luang, Pathum Thani, 12120, Thailand

**Keywords:** *Plasmodium*, Serine hydroxymethyltransferase, Localization

## Abstract

**Background:**

Serine hydroxymethyltransferase (SHMT), a pyridoxal phosphate-dependent enzyme, plays a vital role in the *de novo* pyrimidine biosynthesis pathway in malaria parasites. Two genes have been identified in *Plasmodium* spp. encoding a cytosolic SHMT (cSHMT) and putative mitochondria SHMT (mSHMT), but their roles have not been fully investigated.

**Methods:**

The presence of *Plasmodium* SHMT isoforms in the intra-erythrocytic stage was assessed based on their gene expression using reverse transcription PCR (RT-PCR). Localization studies of *Plasmodium* SHMT isoforms were performed by transfection of fluorescent-tagged gene constructs into *P. falciparum* and expressions of fluorescent fusion proteins in parasites were observed using a laser scanning confocal microscope. Genetic targeting through homologous recombination was used to study the essentiality of SHMT in *Plasmodium* spp.

**Results:**

Semi-quantitative RT-PCR revealed the expression of these two genes throughout intra-erythrocytic development. Localization studies using *P. falciparum* expressing fluorescent-tagged SHMT showed that *Pf*cSHMT-red fluorescent fusion protein (*Pf*cSHMT-DsRed) is localized in the cytoplasm, while *Pf*mSHMT-green fluorescent fusion protein (*Pf*mSHMT-GFP) co-localized with Mitotracker™-labelled mitochondria as predicted. The essentiality of plasmodial cSHMT was inferred from transfection experiments where recovery of viable knock-out parasites was not achieved, unless complemented with a functional equivalent copy of *shmt*.

**Conclusions:**

Distinct compartment localizations of *Pf*SHMT were observed between cytoplasmic and mitochondrial isoforms, and evidence was provided for the indispensable role of plasmodial cSHMT indicating it as a valid target for development of novel anti-malarials.

## Background

The rapid emergence of resistance in *Plasmodium falciparum* to nearly all currently used anti-malarials makes control of falciparum malaria a difficult task. Identification of new drug targets for development of new anti-malarials is urgently needed. The malaria parasite lacks thymidine salvage pathway and depends solely on *de novo* pyrimidine synthesis [1,2], in contrast to the human host, which utilizes both *de novo* and salvage pathways. Serine hydroxymethyltransferase (SHMT) is one of three enzymes involved in dTMP cycle, namely, dihydrofolate reductase (DHFR) and thymidylate synthase (TS). SHMT has a pyridoxal phosphate as a cofactor and participates in one-carbon metabolism, in which SHMT converts serine and tetrahydrofolate (THF) to glycine and methylenetetrahydrofolate (MTHF) respectively. SHMT has been investigated as a possible drug target in cancer and microbial therapeutics, particularly as SHMT expression is tightly regulated with DNA replication during cell division and the enzyme catalyzes the rate-limiting step in dTMP synthesis cycle [3-9].

Two forms of SHMT, cytosolic (c) and mitochondrial (m), can be found in eukaryotes [10,11]. Based on DNA sequence search in PlasmoDB, there are two genes encoding SHMT in *Plasmodium* spp*.*: *Plasmodium falciparum* contains PFL1720w (PF3D7_1235600), a previously characterized cSHMT gene (*Pfcshmt*), and PF14_0534 (PF3D7_1456100), a putative gene of mSHMT (*Pfmshmt*). While the enzymatic function of recombinant *Pf*cSHMT has been shown, the heterologously expressed *Pf*mSHMT was found to be inactive [9,12,13]. *Pf*mSHMT has been proposed to function in association with glycine cleavage components [14], but experimental proof has yet to be provided. As for the cytosolic isoform, alignment of amino acid sequences of *Plasmodium* cSHMT with human cSHMT shows an overall 44% homology and 80% similarity at the active site. In contrast to mammalian SHMTs, *Plasmodium* SHMTs can convert D-serine, in addition to its physiological substrate L-serine, to glycine in the folate-dependent reaction [9,15]. Comparison between the crystal structure of human cSHMT and homology model of *Pf*cSHMT has revealed differences at the substrate binding site, which could be exploited for the development of specific anti-malarial inhibitors that do not cross inhibit the human enzymes [16].

Despite several lines of indirect evidence for the essential role of SHMT in malaria parasite growth, there is hitherto a lack of direct demonstration of this notion. Here, the study provides the genetic evidence confirming the two distinct compartmental localization of SHMT isoforms and demonstrates the indispensable role of cSHMT in growth and development of *Plasmodium* parasites.

## Methods

### Chemicals

All chemicals used were of the highest quality commercially available. The sequences of primers are listed in Additional file 1.

### Semi-quantitative analysis of gene expression of SHMT isoforms in *P. falciparum*

Semi-quantitative reverse transcription PCR (semi-quantitative RT-PCR) was employed to measure expression levels of *Pfcshmt* (primers *Bgl*II 5^′^*Pfcshmt* and *Eco*RV 3^′^*Pfcshmt*) and putative *Pfmshmt* (primers *Bgl*II 5^′^*Pfmshmt* and *Kpn*I 3^′^*Pfmshmt*) relative to that of house-keeping gene *Pfα-tubulin-2* (primers *Pfα-tubulin-2*F and *Pfα-tubulin-2*R). Total RNA was extracted from sorbitol-synchronized *P. falciparum* 3D7 strain at ring, early trophozoite, late trophozoite, and schizont stages using TRIzol® reagent (Invitrogen™, California, USA). Contaminating DNA was removed with RNase-free DNase I (New England Biolabs, Massachusetts, USA). cDNA was synthesized using oligo-dT primer and M-MuLV reverse transcriptase (New England Biolabs). PCR amplification was conducted using GoTaq® DNA polymerase (Promega, Wisconsin, USA) and the following thermal cycling conditions: 95°C for 3 minutes; 20 or 25 cycles of 95°C for 30 seconds, 50°C for 30 seconds, and 72°C for 2 minutes; and a final heating step of 72°C for 5 minutes. Amplicons were resolved by 2% agarose gel-electrophoresis, stained with ethidium bromide, and analysed for their intensities with ImageQuant™ Software (Molecular Dynamics, California, USA).

### Plasmid constructions

Plasmids for the study of gene knockout in *Plasmodium berghei* ANKA strain were constructed based on the sequence of pL0017 vector (The Malaria Research and Reference Reagent Resource Center; MR4), which contains *Toxoplasma gondii dihydrofolate reductase-thymidylate synthase* (*Tgdhfr/ts*) and green fluorescent protein gene (*gfp*) expression cassettes for pyrimethamine (PYR) selection and fluorescence detection of transfected parasites. The 553 and 1,018 bp of PCR amplicons, corresponding to 5^′^- and 3^′^*UTR* of *Pbcshmt* (PBANKA_145020) respectively, were produced initially from *P. berghei* genomic DNA (gDNA). The 5^′^*UTR* fragment was inserted into pL0017 at *Hind*III site, while the 3^′^*UTR* fragment was inserted at *Kpn*I and *Sac*II sites respectively. This construct, pL0017_Δ*shmt*, was used in the knockout study. For allelic replacement construct, *gfp* in pL0017_Δ*shmt* was replaced with *Plasmodium vivax cshmt* (*Pvcshmt*; PVX_100730) and named pL0017_(Pv)Δ*shmt*.

Vectors for localization study were modified from the original pSSPF2/*Pf*Hsp60-GFP vector (a gift from Shigeharu Sato, MRC National Institute for Medical Research, UK) [17]. Initially, a short linker encoding 14 amino acids (SASKLGTSRATNNT) was inserted at *Avr*II restriction site using two complementary oligonucleotides (Linker F and Linker R), which resulted in pSSPF2/*Pf*Hsp60-GFP-Link vector. In order to determine the subcellular localization of *Pf*cSHMT in malaria parasite, *gfp* in pSSPF2/*Pf*Hsp60-GFP-Link was replaced with the gene encoding red fluorescent protein DsRed generating pSSPF2/*Pf*Hsp60-DsRed. Then, the coding sequence of *Pfcshmt* was PCR amplified from cDNA and inserted into pSSPF2/*Pf*Hsp60-DsRed replacing a mitochondrial targeting sequence of *PfHsp60* at *Bgl*II and *Kpn*I sites. For construction of the vector to enable study of putative *Pf*mSHMT localization, human *dhfr* in pSSPF2/*Pf*Hsp60-GFP-Link was replaced with *blasticidin S deaminase* (*bsd*) at *Bam*HI and *Hind*III sites, after which the open reading frame region of putative *Pfmshmt* was inserted at *Bgl*II and *Kpn*I sites. DNA sequences of the two constructs, named pRL_*Pf*cSHMT and pGL_*Pf*mSHMT, were confirmed by DNA sequencing (1st BASE, Singapore).

### Parasite culture and transfection

All animal experiments were performed according to the international and national guidelines for ethical conduct on the care and humane use of animals with approval of the Ethical Committee on Animal Experimentation, National Center for Genetic Engineering and Biotechnology (BIOTEC), Thailand. Mouse strain ICR was intraperitoneally infected with *P. berghei* (10^6^ infected (i) RBC), and blood from tail vein was collected for determining parasitaemia.

Transfection of plasmids into *P. berghei* was performed according to a previously described protocol [18]. In brief, 5–10 μg of each construct were linearized by digestion with *Sac*II, and transfected into purified schizonts using Basic Parasite Nucleofector Kit 2 (Lonza AG, Cologne, Germany) and Amaxa Nucleofector™ device (Amaxa Biosystems GmbH, Cologne, Germany) according to pre-set U033 program. Transfected parasites then intravenously injected into mice tail vein and selected by providing the mice with 70 μg/ml PYR (Sigma-Aldrich, Missouri, USA) in drinking water.

For localization studies, *P. falciparum* strain 3D7 was cultured in human RBC (5% haematocrit) in RPMI-1640 medium (Invitrogen™) supplemented with 0.3 g/l L-glutamine, 5 g/l hypoxanthine and 10% human serum under an atmosphere of 1% O_2_ and 5% CO_2_[19]. Transient transfection of *P. falciparum* with plasmids was performed by electroporation as previously described [20]. In short, 5-10% synchronous ring stage parasites were electroporated with 100 μg of plasmid using Gene Pulser Xcell Electroporation System (Bio-Rad Laboratories, California, USA) at 0.310 kV and 950 μF. pRL_*Pf*cSHMT and pGL_*Pf*mSHMT transfected parasites were cultured in the presence of 2 nM WR99210 and 2 μg/ml blasticidin S (Invitrogen™) respectively.

### Molecular characterization of transfected *P. berghei*

Blood from transfected *P. berghei*-infected mice was collected by heart puncture. White blood cells were removed by passage through a syringe packed with Whatman® CF11 cellulose powder. gDNA was extracted from intra-erythrocytic transfected parasites using Genomic DNA Mini Kit (Geneaid Biotech, Taiwan), and was used in PCR and Southern blotting to assess integration of plasmid constructs at the desired loci. Diagnostic PCR to amplify endogenous *Pbcshmt*, 5^′^ integration fragment, and 3^′^ integration fragment was performed using primer pairs of *Xho*I *Pbcshmt* F and *Bam*HI *Pbcshmt* R, 5^′^UTR int *Pbcshmt* F and 5^′^UTR int *Pbdhfr-ts* R, and 3^′^intDS F and 3^′^UTR int *Pbcshmt* R, respectively. The protocols described above were conducted also with wild type parasites. The putative *Pbmshmt* was amplified as a control (using *Xho*I putative *Pbmshmt* F and *Bam*HI putative *Pbmshmt* R primers).

For Southern blot hybridization, approximately 20 μg of gDNA extracted from transgenic and wild type parasites were digested with *Eco*RV and *Bgl*II. DNA fragments were separated by 1% agarose gel-electrophoresis and transferred to nylon membrane (Merck Milipore, Massachusetts, USA) for hybridization with digoxigenin-labelled 5^′^UTR and 3^′^UTR probes of *Pbcshmt* according to the manufacturer’s protocol (DIG High Prime DNA Labeling and Detection Kit II; Invitrogen™).

Expression of *Pbcshmt*, putative *Pbmshmt*, *Pvcshmt*, and *Pbα-tubulin-2* in wild type and transgenic *P. berghei* parasites were assessed by RT-PCR. Expression level of putative *Pbmshmt* was measured by quantitative RT-PCR (qRT-PCR) using CFX96™ Real-Time System and iQ™ SYBR® Green Supermix (Bio-Rad Laboratories) normalized to *Pbα-tubulin-2* expression level Relative gene expression using 2^-ΔΔCT^ formula. Total RNA and first strand cDNA were prepared for analysis by RT-PCR and qRT-PCR as described above.

### Parasite growth study

Three ICR mice per group were injected intravenously in the tail vein with either wild type or transgenic parasites (10^6^ iRBC/mice). Parasite numbers were counted every day using Giemsa-stained blood smears (from tail vein) under a light microscope.

### Fluorescence microscopy

Parasites were stained with Mitotracker™ (Roche, Basel, Switzerland) and Hoechst 33258 (Sigma-Aldrich) dyes according to manufacturer’s protocols. Localization of fluorescent protein-tagged SHMT isoforms in transfected parasites was determined using Zeiss LSM 700 laser scanning confocal microscope (Carl Zeiss Micro-Imaging GmbH, Germany) at excitation and emission wavelengths of 555 nm and 572 nm respectively for DsRed, and at 488 nm and 509 nm respectively for GFP. Images were processed using ZEN 2009 software.

## Results

### Expression pattern of *Pf*SHMT

**Figure 1:**
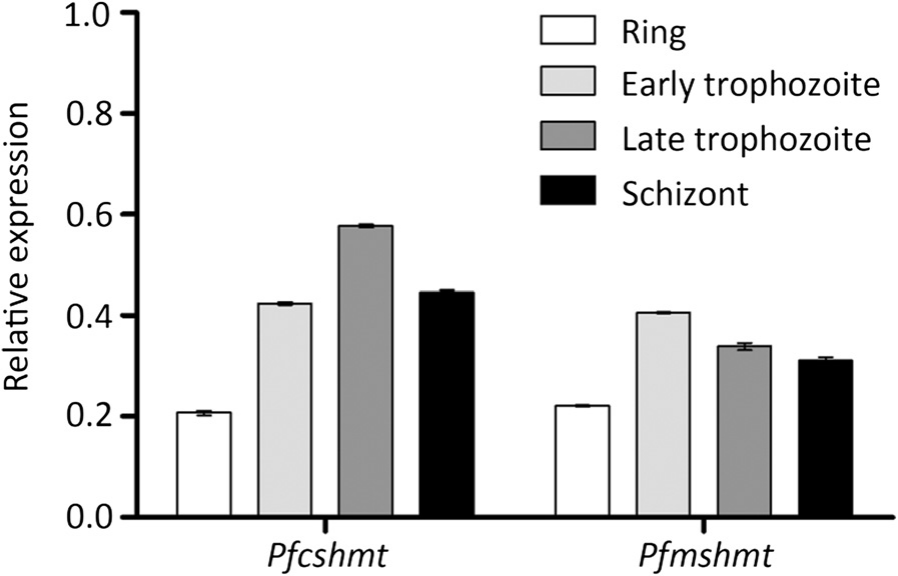
**Expression of *Pfcshmt *and *Pfmshmt *at different intra-erythrocytic developmental stages.** Expressions levels of *Pfcshmt* and *Pfmshmt* transcripts were assessed by semi-quantitative RT-PCR of cDNAs prepared from sorbitol-synchronized *P. falciparum* 3D7 strain. Results are reported as relative values normalized to *Pfα-tubulin*-*2* transcripts.

Although *Pf*cSHMT has been characterized in a number of reports [8,9,12,13,21], not much is known regarding the properties of putative *Pf*mSHMT, except for its mitochondrial location based on gene prediction and annotation in PlasmoDB. When the expression patterns of the two *shmt* forms were compared in *P. falciparum* during intra-erythrocytic developmental stages using semi-quantitative RT-PCR (normalized to that of *Pfα-tubulin-2* expression), transcripts of both *Pfshmt* forms were detected throughout all erythrocytic stages, with peak transcript levels appearing during early and late trophozoite stage for putative *Pfmshmt* and *Pfcshmt* respectively (Figure 1). These results are in agreement with the role of SHMT in dTMP biosynthesis required for DNA replication.

### Localization of SHMT isoforms in *P. falciparum*

**Figure 2:**
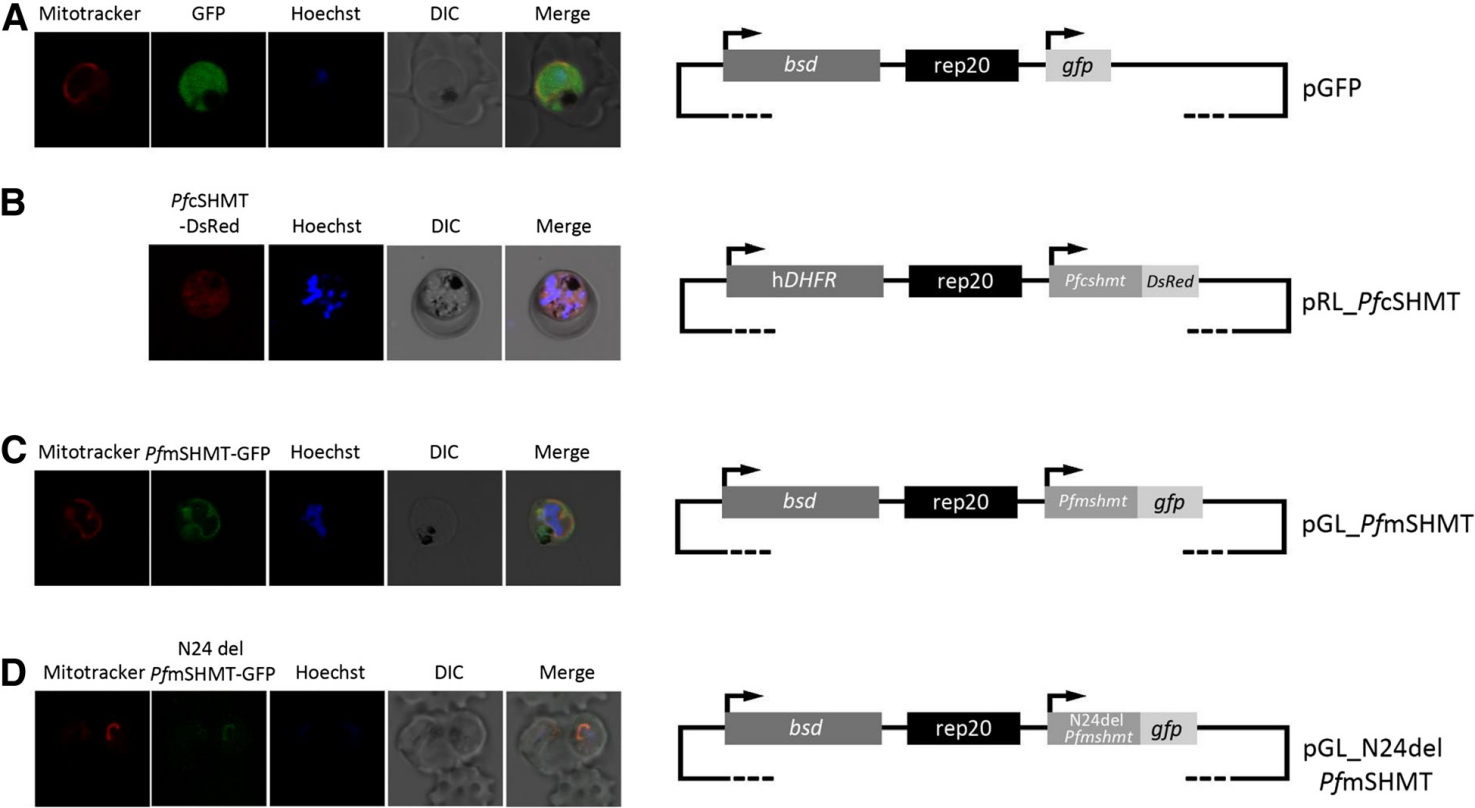
**Localization of *P. falciparum *SHMT isoforms.** Parasites were transfected with pGFP (**A**), pRL_*Pf*cSHMT (**B**), pGL_*Pf*mSHMT (**C**), and pGL_N24del *Pf*mSHMT (**D**) plasmids expressing fluorescent signals from GFP or DsRed. Schematic diagrams of the recombinant plasmids used are shown alongside the confocal micrographs. Mitochondrion and nucleus is stained with Mitotracker™ (red) and Hoechst 33258 (blue) dye respectively. DIC, differential interference contrast image; rep20, rep20 sequence; *bsd*, *blasticidin S deaminase*; h*DHFR*, human *dihydrofolate reductase*; *gfp*, *green fluorescence protein*; *DsRed*, *red fluorescent protein*.

In order to identify the locations of the two *Pf*SHMT isoforms in the parasite, pRL_*Pf*cSHMT and pGL_*Pf*mSHMT plasmids were constructed to express *Pf*cSHMT fused with DsRed and *Pf*mSHMT fused with GFP in the parasites (Figures 2B and 2C). The expression of these fusion proteins is driven by the constitutive promoter, *Pf*Hsp86 [17,22,23]. Transgenic parasites expressing GFP alone showed a diffuse distribution pattern of green fluorescence throughout the cytoplasm (Figure 2A). The distribution of DsRed-tagged *Pf*cSHMT appears to be dominantly in cytoplasm (Figure 2B). *Pf*cSHMT-DsRed fluorescence appears more variable in intensity compared to that of GFP alone in the cytoplasm. This may be partly explained by the superior fluorescence properties of GFP which is more intense and photostable compared to those of DsRed [24]. The intensity of the fluorescence reporter protein may also be affected when fused with another protein. The distribution of GFP-tagged *Pf*mSHMT was co-localized with Mitotracker™ within the mitochondria (Figure 2C). The micrograph also suggest less intense signal seen in the area conterminous with the food vacuole, which likely comes from auto-fluorescence of crystalline haemozoin. This phenomenon was also observed in non-transfected parasites, especially when the image brightness was enhanced.

In most eukaryotes, a mitochondrial localization signal sequence is located at the N-terminus of proteins targeted to this organelle [25]. However, this feature is not well-characterized in *Plasmodium*. PlasMit program [26] is the only available tool to predict *Plasmodium* mitochondrial-targeted proteins based on previously characterized malaria parasite mitochondrial proteins, which analyzed the first 24 N-terminal amino acids of *Pf*mSHMT and predicted it to be a mitochondrial protein. Plasmid pGL_N24del *Pf*mSHMT, expressing GFP-tagged *Pf*mSHMT with deletion of the first 24 N-terminal amino acids (N-24 truncated *Pf*mSHMT-GFP), was constructed to test the validity of this sequence as mitochondrial targeting signal. Confocal microscopy showed co-localization of N-24 truncated-*Pf*mSHMT-GFP with Mitotracker™ (Figure 2D), indicating that this 24 N-terminal amino acid sequence does not play a role as a mitochondria targeting signal.

**Figure 3:**
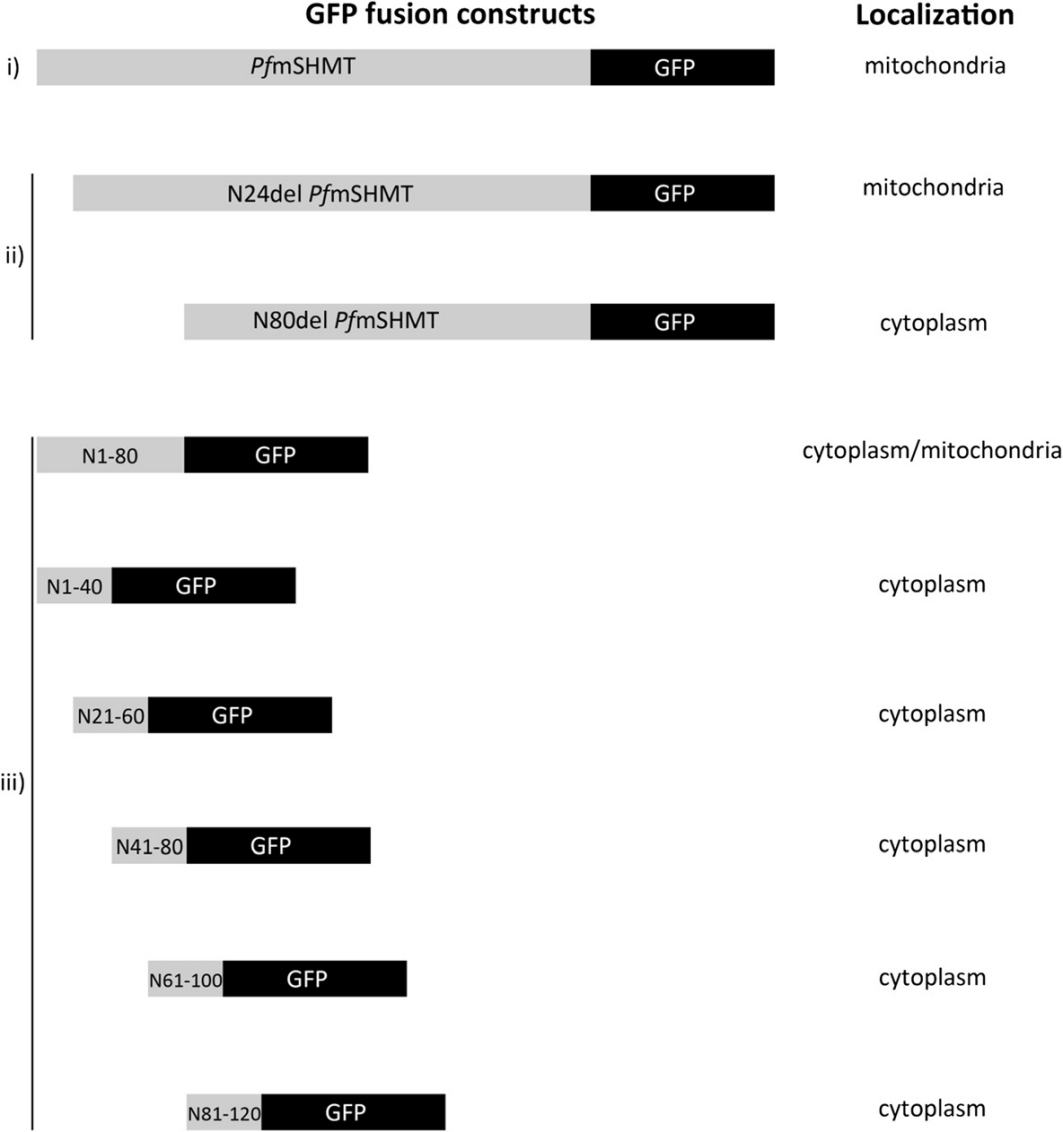
**Mitochondrial signal peptide mapping of *Pf*mSHMT.** Schematic diagrams depict localization of GFP fusion protein with (**i**) full-length *Pf*mSHMT, (**ii**) N-terminus truncated *Pf*mSHMT, and (**iii**) a series of truncated N-terminal 1–120 amino acid fragment of *Pf*mSHMT. N24del and N80del refer to deletion of N-terminal amino acids at positions 1–24 and 1–80 of *Pf*mSHMT respectively. The numbers in N1-80, N1-40, N21-60, N41-8 0, N61-100 and N81-120 refer to amino acid positions at N-terminus of *Pf*mSHMT.

Recently, Read *et al.*[27] reported that the first 100 N-terminal amino acids of *Pf*mSHMT targets GFP-fusion protein to the mitochondrion. In order to identify the minimal mitochondrion-targeting sequence, plasmids were constructed to express GFP fusion proteins containing a series of truncations of the N-terminal 120 amino acids (Figure 3). All sequence-tagged-GFP were found only in the cytoplasm, except N1-80-GFP that was present in both mitochondrion and cytoplasm. Along with these results, in a separate experiment, the truncated del-N1-80-*Pf*mSHMT-GFP was found in cytoplasm. The data suggest that the mitochondrial signal sequence is likely to involve longer span of *Pf*mSHMT N-terminal amino acids than previously predicted.

### Role of cSHMT in *Plasmodium* erythrocytic stages

**Figure 4:**
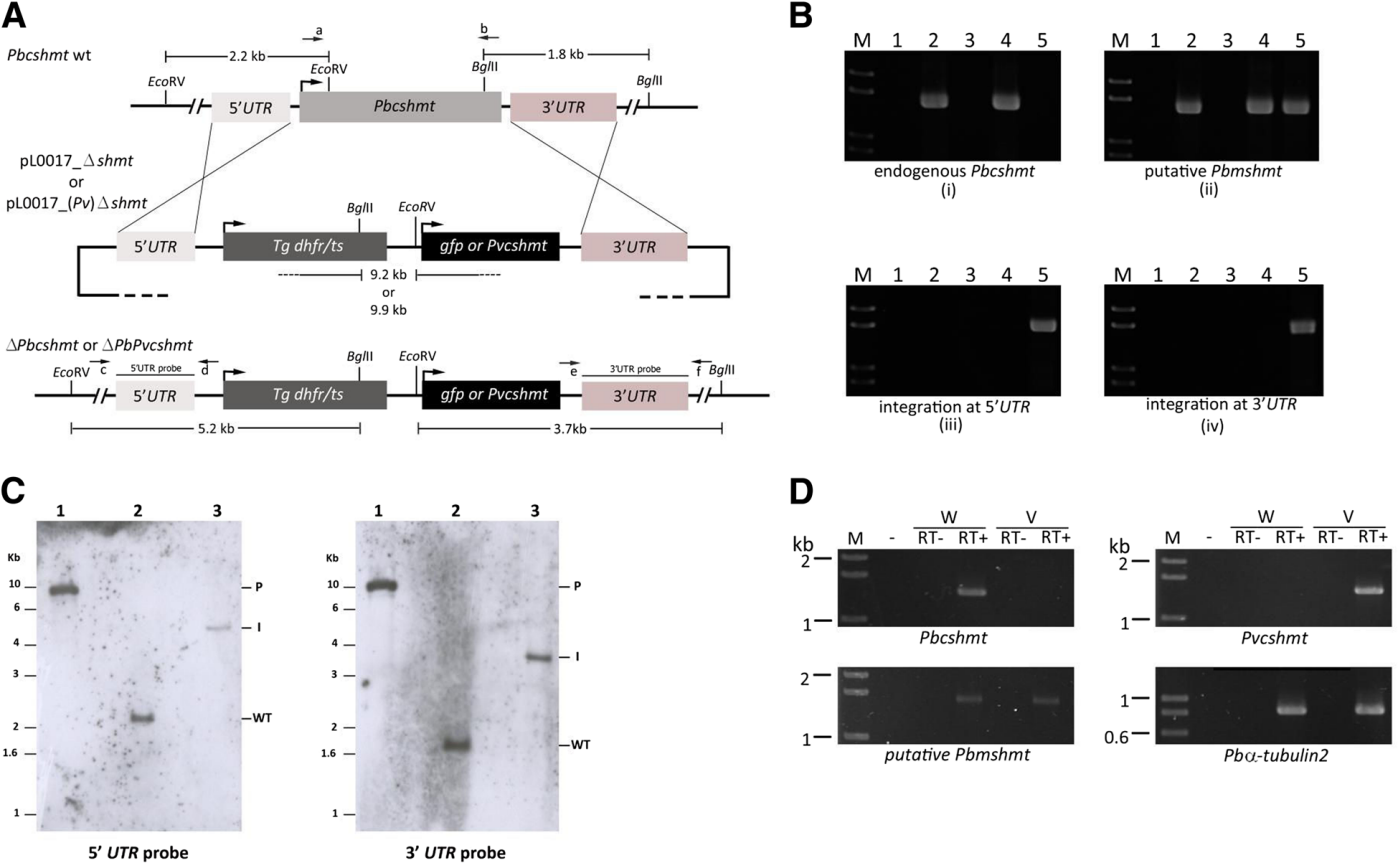
**Targeted deletion of *Pbcshmt*.** (**A**) Schematic diagram depicting the genomic organization of *Pbcshmt* locus following disruption or allelic replacement with *Pvshmt* coding sequence. Enzyme restriction sites, along with fragment sizes and their specific probes are indicated. (**B**) PCR diagram of molecular characterization of transfected parasites. Lanes 1–5: (1) water control; (2) gDNA of *P. berghei* wild type; (3) pL0017_(Pv)Δ*shmt*; (4) and (5) gDNA of *P. berghei* transfected with pL0017_Δ*shmt* and pL0017_(Pv)Δ*shmt,* respectively. Primer pairs a & b, c & d, and e & f (sequences reported in Additional file 1) are used to amplify (i) endogenous *Pbcshmt*, (iii) 5^′^ integrated fragment, and (iv) 3^′^ integrated fragment, respectively. Amplification of putative *Pbmshmt* (ii) was performed as a control. (**C**) Southern blot hybridized with 5^′^ or 3^′^*UTR* probe to confirm *Pvcshmt* allelic replacement at *Pbcshmt* locus. DNA was digested with *Eco*RV and *Bgl*II. Lanes are: (1) pL0017_(Pv)Δ*shmt* plasmid, (2) gDNA of *P. berghei* wild type, and (3) gDNA of transgenic Δ*PbPvcshmt P. berghei*, respectively. P, I, and WT indicate expected band size for pL0017_(Pv)Δ*shmt* plasmid, integrated *Pvcshmt*, and endogenous *Pbcshmt,* respectively. (**D**) RT-PCR diagram for detection of *shmt* transcript. Lanes are: (M) 1kb ladder, (−) water control, (RT-) no RT control, (RT+) cDNA, (W) *P. berghei* wild type, and (V) transgenic *P. berghei* harbouring *Pvcshmt*. *Pbmshmt* and *Pbα-tubulin-2* were amplified as control genes.

The role of *Plasmodium* cSHMT was assessed using *P. berghei* transfection system because genetic manipulations of *P. berghei* are more efficient than that of *P. falciparum*[28]. Two transfection plasmids were constructed, which upon double crossing-over recombination event from transfecting with linearized plasmids, resulted in replacement of *Pbcshmt* with the coding sequence either *Pvcshmt* or *gfp* (Figure 4A). Following selection of transfected *P. berghei* with PYR, only transgenic parasites containing *Pvcshmt* in place of *Pbcshmt* were recovered (Δ*PbPvcshmt*). These results were consistent in three independent experiments. DNA analysis of transgenic parasite clones (obtained by limiting dilution) was undertaken to confirm the presence of the transgene, *Pvcshmt*. PCR amplification using specific primers for the recombination events in transgenic parasites showed a set of unique bands indicative of the integration of *Pvcshmt* (Figure 4B). Southern blot analysis indicated an absence of endogenous *Pbcshmt* in *Pvcshmt* replacement clones (Figure 4C). In addition, RT-PCR confirmed the absence of *Pbcshmt* expression, but that of *Pvcshmt* transcript instead (Figure 4D). These results rule out the recombination refractory property of the *Pbcshmt* locus and lend support to the indispensability of *shmt* for malaria parasite growth and development.

### Expression level of *shmt* in transgenic rodent malaria parasites and effect on growth rate

**Figure 5:**
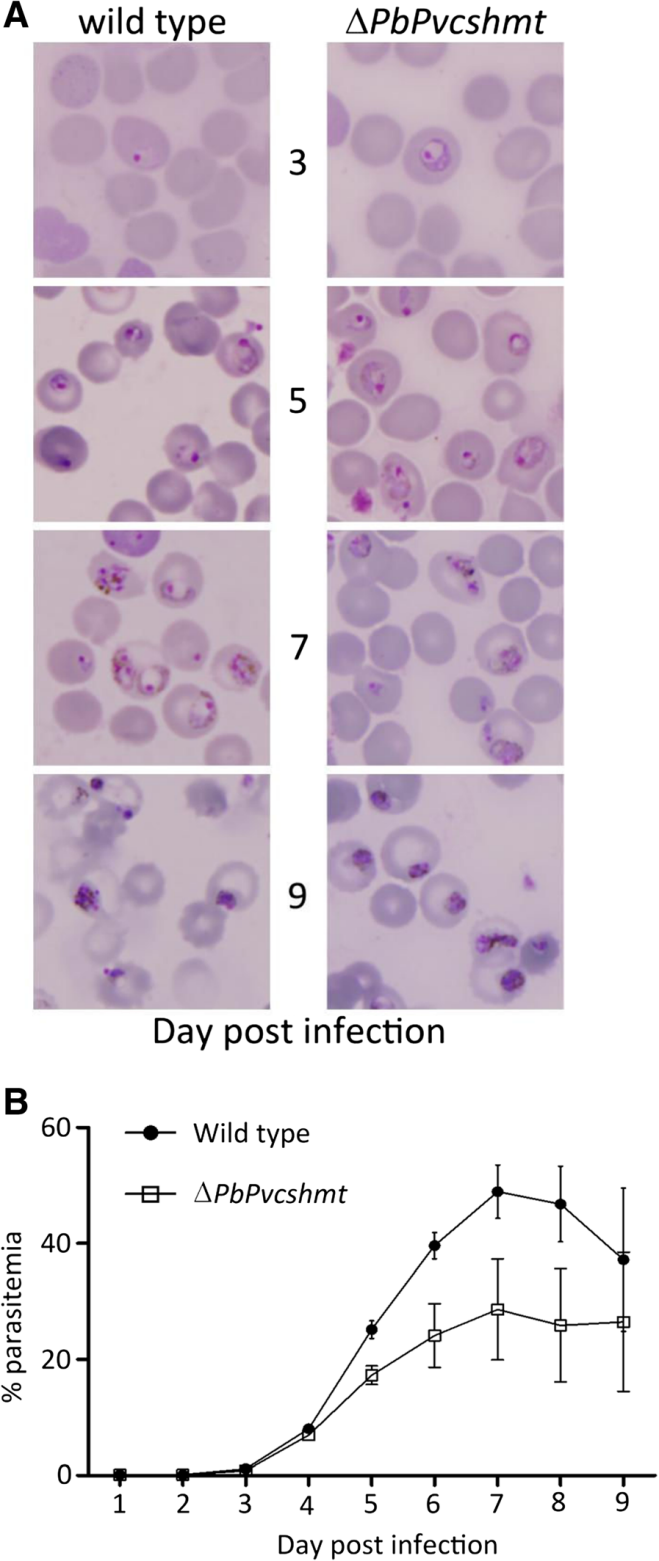
**Morphology and parasite count.** Morphology (**A**) and parasite count (**B**) of wild type and Δ*PbPvcshmt* (allelic replacement with *Pvshmt* coding sequence) transgenic *P. berghei*. Thin blood smear and parasitaemia determination were performed every day post infection for 9 days.

In order to examine if the presence of *Pvcshmt* in transgenic *P. berghei* is functionally equivalent to that of endogenous *Pbcshmt*, growth profiles of these parasites were compared. Morphology of transgenic Δ*PbPvcshmt* parasites at different sampling times did not appear to be affected (Figure 5A). However, the parasitaemia of transgenic Δ*PbPvcshmt* parasites appeared to be less than that of the wild type (Figure 5B).

A redundant role for the two SHMT isoforms has been demonstrated in eukaryotes [29]. On the other hand, attempts in this study to knock out *cshmt* from both *P. falciparum* and *P. berghei*, and leaving *mshmt* intact, were unsuccessful. Expression profiles of *mshmt* in wild type and transgenic Δ*PbPvcshmt* parasites were comparable*.* Thus, it does not appear that there is a redundancy role between plasmodial c- and mSHMT.

## Discussion

SHMT links together several metabolic pathways, including biosynthesis of folate, dTMP, and methionine. The biological necessity of this enzyme in malaria parasites has been proposed as *shmt* transcripts are markedly increased during the rapid intra-erythrocytic stage progression [8]. Similar to other eukaryotes, *Plasmodium* spp. has two SHMT isoforms, a functioning *cshmt* and a putative *mshmt* allele. It is worth noting that, unlike other eukaryotes where c- and m-SHMT isozymes are highly conserved, *Pf*cSHMT and *Pf*mSHMT share only ~20% similarity with each other [30].

Here, results show that *Pfmshmt* is a functional gene by demonstrating the expression of gene product throughout the asexual stage development. The presence of two isoforms in *Plasmodium* spp. raises the possibility of a redundant role and a potential overlap in their functional activity. For instance, in mice, examination of nuclear extracts of *cshmt-*knockout mice showed 25% SHMT activity compared to wild type mice; the remaining SHMT activity is due to the presence of mSHMT in the nuclear extract, suggesting a redundant function of the two murine SHMT isoforms [29]. In the case of *Plasmodium* spp., null-mutants of *cshmt*-knockout parasite clones could not be recovered from transfected *P. falciparum,* even though methionine, folinic acid, or a mixture of these compounds was supplemented at concentrations 10-fold higher than that present in RPMI. However, in *P. berghei*, *cshmt*-knockout parasite clones could only be recovered when complemented with *cshmt* from another *Plasmodium* species (in this case *P. vivax*). These results provide experimental confirmation of the essentiality of *cshmt* in the survival of malaria parasites. In addition, these results suggest that there is functional conservation of cSHMT among *Plasmodium* spp., but not between cSHMT and mSHMT of the same species. On-going efforts to express recombinant mSHMT are in progress in order to confirm its role in malaria parasites.

The first 24 N-terminal amino acids of putative plasmodial mSHMT contain several basic amino acids characteristics of mitochondrial targeting sequence [26,31]. Previous study observed that the first 100 N-terminus of *Pf*mSHMT is sufficient for mitochondria targeting [27]. In this study, transfection system using GFP reporter gene was taken to examine the cellular localization of *Pf*mSHMT and to identify the minimum sequence required for mitochondrial targeting of this enzyme. Contrary to the previous prediction, the removal of putative mitochondria signal sequence (N-terminus amino acids 1–24) of *Pf*mSHMT did not affect its localization to the mitochondria, suggesting that that the targeting sequence may be downstream of the putative mitochondria targeting sequence. Systematic deletions of the first 120 amino acids of *Pf*mSHMT demonstrated that the minimum leader sequence for mitochondrial targeting lies between amino acids 25–80. However, detection of cytoplasmic/mitochondria fluorescence of N1-80-GFP suggests that a more complex mechanism may be involved, such that a longer signal sequence may provide more specific localization to the mitochondria.

Intracellular localizations of *Pf*cSHMT and *Pf*mSHMT were addressed in this study by direct observation of SHMTs fusion with reporter protein compared to previously published work using immunofluorescence approach (IFA) [27]. The IFA with polyclonal antibody suggested a stage dependent localization pattern where *Pf*cSHMT appeared in the cytoplasm, and also to apicoplast in the mid/late trophozoite to schizont stage. *Pf*mSHMT appeared mainly in the mitochondria with some distribution in the cytoplasm. Multi-organelle localizations observed in these IFA experiments may be in part due to cross-reaction of polyclonal antibodies. Whilst the current work relies upon the intrinsic fluorescence from GFP or DsRed fused to SHMT of interest, with the assumption that the fusion proteins behave the same as native SHMTs. Despite different approaches, these studies are complementary of each other, as both studies revealed distinct compartment localization of *Pf*cSHMT and *Pf*mSHMT.

Various phenotypic consequences in *shmt*-deficient cells have been described. Inactivation of *shmt* results in glycine auxotroph phenotype in some organisms, such as *Escherichia coli*[32], while *shmt* mutations in *Caenorhabditis elegans* lead to maternal-effect lethal phenotype [33], pointing to the essential role of SHMT. In this study, attempts were made to generate *Pbcshmt* null mutant but the gene could not be replaced by a knockout construct. The refractoriness of *Pbcshmt* locus was ruled out as our attempts to replace the endogenous gene with *Pvcshmt* were successful. Additionally, the redundancy role of SHMTs in malaria parasite can be excluded. Transgenic *P. berghei* parasites containing *Pvcshmt* were able to infect murine red blood cells and complete their blood stage life cycle, albeit at a lower parasitaemia when compared with that of the wild type parasites. This implies that replacement of *shmt* affects fitness of transgenic parasite, which may be due to differences in catalytic efficiency between rodent and human plasmodial enzymes. This could readily be proven by comparing kinetic parameters of recombinant *Pb*cSHMT and *Pv*cSHMT. It should also be noted that the expression of *Pv*cSHMT was regulated by *Pb*eef1α promoter, which might have an effect on the growth of mutant parasite.

*Plasmodium* SHMT has been suggested to be the rate-limiting enzyme in dTMP synthesis pathway [8], and thus is a potential target for drug development. Various classes of compounds, including 2,4-diaminopyrimidine, have been proposed to be effective inhibitors of *Plasmodium* SHMT based on binding affinity obtained from molecular docking calculations [34,35]. The recent study has shown that a number of 2,4-diaminopyrimidine compounds can inhibit *Plasmodium* SHMT [21]. Further optimization employing a target-based design approach should allow design of more effective anti-malarial drugs targeting *Plasmodium* SHMT.

## Conclusions

Both isoforms of plasmodial SHMT are highly expressed during the trophozoite stage, which highlight the role of these enzymes during parasite growth and development. Fluorescent-tagged plasmodial SHMT proteins confirmed the expected sub-cellular location of cytoplasmic and mitochondrial SHMT. The indispensable role of *Plasmodium* cSHMT for intra-erythrocytic development was inferred from the inability to generate *cshmt* knock-out parasites, which also indicated that the two SHMT isoforms do not possess redundant function typical of other eukaryotes. Therefore, cSHMT is a validated anti-malarial drug target. Furthermore, transgenic *P. berghei* containing human malaria *cshmt* in place of endogenous gene will serve as an *in vivo* model for evaluation of novel anti-malarials directed against human plasmodial SHMT.

## Abbreviations

SHMT: Serine hydroxymethyltransferase; DHFR: Dihydrofolate reductase; TS: Thymidylate synthase; MTHF: Methylenetetrahydrofolate; THF: Tetrahydrofolate; c: Cytosolic; m: Mitochondrial; Pf: *Plasmodium falciparum*; Pb: *P. berghei*; Pv: *P*. *vivax*; 3^′^UTR: 3^′^ untranslated region; 5^′^UTR: 5^′^ untranslated region; BSD: Blasticidin S deaminase; PYR: Pyrimethamine; RBC: Erythrocyte; iRBC: Infected erythrocytes; DsRed: *Discosoma sp.* Red fluorescent protein; gDNA: Genomic DNA.

## Competing interests

The authors declare that they have no competing interests.

## Authors’ contributions

WP performed the study and drafted the manuscript. DK participated in the design of the study and drafted the manuscript. CU and YY discussed and commented on the manuscript. UL conceived of the study, and participated in its design and coordination and helped to draft the manuscript. All authors read and approved the final manuscript.

## Supplementary Material

Additional file 1 Primers used in the study. Click here for file
